# The Global Fund: why anti-corruption, transparency and accountability matter

**DOI:** 10.1186/s12992-021-00753-w

**Published:** 2021-09-18

**Authors:** Zhihao Chang, Violet Rusu, Jillian C. Kohler

**Affiliations:** 1 Leslie Dan Faculty of Pharmacy, University of Toronto, Toronto, Ontario Canada; 2 WHO Collaborating Centre (WHO CC) for Governance, Accountability and Transparency in the Pharmaceutical Sector, Leslie Dan Faculty of Pharmacy, University of Toronto, Toronto, Ontario Canada; 3 Dalla Lana School of Public Health, University of Toronto, Toronto, Ontario Canada

**Keywords:** Anti-corruption, Transparency, Accountability, ACTA, Global Fund, GFATM, The fund

## Abstract

**Background:**

The creation of the Global Fund to Fight AIDS, Tuberculosis and Malaria, also known as the Global Fund, was prompted by the lack of a timely and effective global response, and the need for financing to fight against three devastating diseases: HIV/AIDS, tuberculosis, and malaria. During the formation of the Global Fund, necessary anti-corruption, transparency, and accountability (ACTA) structures were not put in place to prevent fraud and corruption in its grants, which resulted in the misuse of funds by grant recipients and an eventual loss of donor confidence in 2011. The Global Fund has instituted various ACTA mechanisms to address this misuse of funding and the subsequent loss of donor confidence, and this paper seeks to understand these implementations and their impacts over the past decade, in an effort to probe ACTA more deeply.

**Results:**

By restructuring the governing committees in 2011, and the Audit and Finance; Ethics and Governance; and Strategy Committees in 2016, the Global Fund has delineated committee mandates and strengthened the Board’s oversight of operations. Additionally, the Global Fund has adopted a rigorous risk management framework which it has worked into all aspects of its functioning. An Ethics and Integrity Framework was adopted in 2014 and an Ethics Office was established in 2016, resulting in increased conflict of interest disclosures and greater considerations of ethics within the organization. The Global Fund’s Office of the Inspector General (OIG) has effectively performed internal and external audits and investigations on fraud and corruption, highlighted potential risks for mitigation, and has implemented ACTA initiatives, such as the *I Speak Out Now!* campaign to encourage whistleblowing and educate on fraud and corruption.

**Conclusions:**

From 2011 onwards, the Global Fund has developed a number of ACTA mechanisms which, in particular, resulted in reduced grant-related risks and procurement fraud as demonstrated by the decreased classification from high to moderate in 2017, and the reduction of investigations in 2018 respectively. However, it is crucial that the Global Fund continues to evaluate the effectiveness of these mechanisms; monitor for potential perverse impacts; and make necessary changes, when and where they are needed.

## Background

The Global Fund to Fight AIDS, Tuberculosis and Malaria, also known as the Global Fund, was established in 2002 as an international financial institution to pool donations and rapidly disburse grants to help in-need countries procure medicines as a part of the global response to the HIV epidemic [1]. The Global Fund was created in response to a clear need in the global health space for emergency financing to fight three major infectious diseases: HIV/AIDS, tuberculosis, and malaria, which continue to kill more than 2.4 million people per year [2, 3]. Since its inception, the Global Fund has proven to be a critical global health organization, providing 18.9 million people with antiretroviral therapy, 5.3 million people with tuberculosis treatment, and as of 2018, helping to save 32 million lives globally [4].

Still, the Global Fund has had to overcome corruption and fraud in the implementation of its grants, partially due to its failure to enact necessary anti-corruption, transparency, and accountability (ACTA) mechanisms during the organization’s formation [5]. Corruption, defined by Transparency International as, “*the abuse of entrusted power for private gain*,” is found in health care systems globally and has a negative effect on the security, population health, development, and economic growth of society [6–8]. Corruption in health is difficult to measure as it is often hidden and can sometimes be attributed to administrative inefficiencies. However, it is estimated that each year $455 billion of the annual $7.35 trillion spent on health care globally is lost to corruption and fraud [8, 9]. Garcia (2019) refers to corruption as an “ignored pandemic,” one that claims the lives of 140,000 children annually [10].

The impact of corruption can be deadly, with challenges that are further exacerbated in the context of global health [10]. Global health is particularly vulnerable to corruption as it is “multifaceted and complex” in nature, often including more than one country and multiple development partners [8]. Corruption has a global reach; it can manifest in many forms across various types of institutions, fuelling inequity and working to undermine global health efforts such as universal health coverage and other health targets designated in the UN’s Sustainable Development Goals (SDGs) [8, 11].

The Global Fund’s original grant disbursement framework was based around the goal of providing support to national strategies in a simple, rapid, and innovative manner that prioritized the countries most affected—a goal which it is argued they have since deviated from [12]. During several country audits, the Global Fund’s Office of the Inspector General (OIG) discovered various cases of corruption amounting to levels referred to as “astonishing” by the Associated Press (AP), such as the misuse of US$ 34 million in funding by four beneficiary countries [13, 14]. Based on information detailed in the OIG country audit reports, the AP publicized the fraud and corruption cases, including tens of millions of dollars in annual shipments of malaria medication that were stolen for resale and millions of dollars lost to fraud, misappropriated funds, false documentation, ineligible costs, and funds unaccounted for in various countries [13]. Despite report findings, the Global Fund responded to the AP article, contending that their corruption problems were no more serious than any other international organization [13]. Nonetheless, in 2012, the Global Fund entered survival mode after it was forced to cancel its funding round due to decreased pledges from donors amidst the global economic crisis and the publicized reports of corruption [12].

Prior to the AP article, there were previous calls for transparency and mutual accountability within the Global Fund [15]. Additionally, donor agencies have received criticism for not being selective enough with the countries aid is provided to. According to Radelet (2004), the Global Fund has operated in a manner that seems to allocate aid heavily based on the level of need, although it does not have an official policy to do so: “In its first two years GFATM has generally approved larger grants for countries with higher disease burdens … the Fund has rejected the ‘country selectivity’ idea: it has not approved more money for countries with better governance and stronger overall development policies, or less money for those with higher levels of corruption or a weak policy commitment” [16]. Sidibe et al. (2006) explain that based on lessons learnt within the Global Fund, procedures for mutual accountability and transparency were necessary to ensure the organization’s credibility and the effectiveness of its funding [15]. Additionally, after its first five-year evaluation, warnings went ignored when the Global Fund received comments of unrealistic program expectations that could lead to future problems [12].

The Global Fund’s publicly exposed challenges with corruption and fraud in its funding disbursements led to a significant loss of donor confidence in 2011, with Sweden, Germany, Denmark, and the European Commission all freezing their funding until the organization demonstrated it was addressing these vulnerabilities [17, 18]. This compelled the Board of the Global Fund to establish a “High-Level Independent Review Panel” (HLP), which would “review and evaluate the Global Fund’s related policies, procedures, practices, control systems, and oversight mechanisms” to assess their effectiveness and susceptibility to fraud [14]. In 2011, the HLP’s final report made six recommendations: that the Global Fund transition to a sustainable response opposed to emergency; create a new strategy for risk management; enhance governance internally; launch a new process for grant approvals; improve decision making processes in management; and “get serious about results” [19]. The panel also noted that the Global Fund risked withering if it didn’t implement changes [19].

Since establishing the HLP, the Global Fund has reworked its governance structures and implemented various ACTA mechanisms, including making improvements to the pre-existing OIG, to address the misuse of funding and the subsequent loss of donor confidence in 2011. This paper seeks to understand these implementations and their impacts over the past decade, in an effort to probe ACTA more deeply. While the focus of this paper is on the ACTA mechanisms implemented from 2011 onwards, the OIG is included in the scope of this paper as it has implemented new initiatives and made constant improvements based on recommendations from the HLP.

## Methods

This paper is a follow-up to a broader exploration of the ACTA mechanisms within international organizations including the World Health Organization (WHO), the World Bank Group, the UNDP, and the Global Fund, with the latter of higher significance to this paper [18]. This paper builds upon previous findings by providing an in-depth description and analysis of the specific internal ACTA mechanisms employed by the Global Fund. Our methods consisted of the following: firstly, the organizational structure of the Global Fund, its ACTA mechanisms, and its investigations unit were reviewed. Following this, the most current documents, policies and reports that were found on the websites of the Global Fund and its Office of the Inspector General as of April 7, 2020, were also reviewed. Further searches on the Global Fund’s website were guided by key terms used in those documents. A literature review was also performed on SCOPUS, PubMed, EMBASE, and MEDLINE databases with the key search term “‘Global Fund’ AND (corruption OR fraud)” on April 7, 2020, without date restriction. This search revealed 11 hits on SCOPUS, 10 hits PubMed, nine hits on EMBASE, and eight hits on MEDLINE. After removing duplicates, 11 unique articles and relevant papers were selected based on a screening of the abstract for corruption and fraud within the Global Fund and its grant beneficiaries. These searches also led to the discovery of the 2011 Associated Press article and the Global Fund’s Recoveries Reports. The same search term on Google led to the Aidspan website, an independent observer of the Global Fund. Although the Aidspan website was consulted for relevant information, it is an independent organization and therefore its operations are outside the scope of this paper. Additionally, a hand search of relevant academic articles was conducted, from which 21 articles were selected for inclusion.

## Results

The Global Fund has implemented significant mechanisms to increase ACTA within the organization since the loss of donor confidence in 2011, including making improvements to the pre-existing OIG. The Global Fund alleges that it is committed to “zero tolerance to corruption” and recognizes that corruption and fraud “corrode public health institutions and systems and facilitate human rights abuses, ultimately stunting the quality and quantity of interventions needed to save lives” [20, 21]. Even though it is an aspirational commitment, efforts have been made by the Global Fund to work towards it.

According to the Global Fund’s Framework Document, the organization promotes country accountability while integrating multiple levels of control, which allows it to minimize an in-country presence [5]. This offers country ownership over grants in the hope that programs will lend “an extensive degree of local control” and lead to an “acceptance of responsibility and accountability for expenditure and results” [5]. More specifically, each country has a Country Coordinating Mechanism (CCM), which composes country-specific grant proposals and submits them to the Global Fund [5]. The CCMs are responsible for fulfilling the “Code of Ethical Conduct for Country Coordinating Mechanism Members,” which was approved in April 2019 and has stipulations regarding anti-fraud and anti-corruption measures, such as restrictions to mitigate conflicts of interest and a requirement to prevent, detect, and stand up to corruption [22].

The Global Fund has also enacted a robust whistleblowing policy, detailed audits and investigations, conflict of interest disclosures, and risk management strategies to mitigate fraud and corruption, which have evolved over time, particularly after the HLP report. The Global Fund’s various ACTA mechanisms are described below, including the Global Fund’s Board of Directors and its executive committees, risk management and ethics within the Global Fund, the OIG and associated audits and investigations, and the Global Fund’s Pooled Procurement Initiative.

### Institutional governance

#### The Board of Directors and its committees

At the highest level of the organization is the Global Fund’s Board of Directors, composed of 20 voting members who meet at least twice per year, with representation from implementing countries, donors, NGOs, and nonvoting members, including USAID, the WHO, the World Bank, and other multilateral organizations [23]. The 2011 HLP report made several evaluations of the Board, identifying that the Committees had overlapping mandates, inconsistent membership, and weak capacities [5]. For example, it claimed that the Finance and Audit Committee was not “optimally effective, because of its lack of technical expertise and failure to respond adequately to, and follow up on, OIG reports” [5].

In 2011, the Board replaced the previous standing committees with the “Strategy, Investment and Impact Committee,” “Finance and Operational Performance Committee,” and “Audit and Ethics Committee.” Additionally, the Committees’ terms of reference were updated to incorporate HLP recommendations, and a coordinating group was created to enhance collaboration and allow for “more empowered management” in providing oversight of governance structures within the Global Fund [24].

The standing committees were restructured once again in January 2016 following a 2014 OIG report which reviewed the effectiveness of the Global Fund’s governance structures. Subsequently, the “Audit and Finance Committee,” the “Ethics and Governance Committee,” and the “Strategy Committee” were formed [25–27]. Once again, the OIG reviewed the Global Fund’s governance structures in 2017 and noted significant improvements. There is now a standing committee responsible for matters of governance, and the coordinating group has started to prioritize issues that span all three Committees [28]. Currently, the Audit and Finance Committee oversees the management of the Global Fund’s financial resources, internal and external audits, and OIG investigations [26, 27]. The Ethics and Governance Committee oversees the governance structures and promotes ethical standards according to the organization’s framework on ethics [26, 27]. Finally, the Strategy Committee oversees the Global Fund’s “strategic direction” [26]. The changes implemented from 2011 onwards have enhanced the Global Fund’s governance structures, which have improved trust and accountability within the organization and have allowed the Board to play a greater role in strategy, financial oversight, risk management, and in leading the organization’s response to fraud and corruption [28].

#### Risk management

Risk management has proven to be an effective preventative approach to corruption in global health organizations and one that the Fund advocates. By putting the focus on prevention rather than penalization, an international organization can identify the possible entry points for corruption and proactively build institutional capacity. Furthermore, taking a preventative approach to corruption allows for public trust-building in the system and shifts the focus away from measures that are reactive, while creating room for new innovations to prevent corruption, such as risk management [29]. Throughout the past decade, the Global Fund has consistently and actively reworked and strengthened its risk management framework, which the HLP identified as a particularly lacking area [5].

Starting in 2011, the Global Fund developed a risk management methodology that assessed the potential impact of different grant-implementation risks and created follow-up action plans for mitigation [30]. The identified risks were classified from low to very high risk and split into broad categories: 1) “Programmatic and Performance Risks,” 2) “Financial and Fiduciary Risks,” 3) “Health Services and Product Risks,” and 4) “Governance, Oversight and Management Risks” [30]. Of particular importance for mitigating fraud and corruption in the implementation of Global Fund grants are the “financial and fiduciary risks.” Using this methodology, risk assessments and action plans are created, and results are subsequently reviewed to create focused mitigation measures [30]. These assessments feed into the “Portfolio Risk Index,” which is a marker of overall risk in the Global Fund Portfolio [31]. This type of approach is not only applied in the Global Fund, but similar risk mitigation strategies are also applied by the WHO and the United Nations Office on Drugs and Crime (UNODC) [32, 33].

In 2012, a “Risk Management Department” and the position of Chief Risk Officer (CRO) were created to coordinate risk management and to develop a common language, strategy and action plan across the Global Fund [30]. The CRO is well integrated into important functions of the organization and is a senior member of management committees and the chair of the Recoveries Committee, which is responsible for recovering funds lost to fraud and corruption [30]. In November 2014, the Global Fund published its risk management policy with the objective to make “risk management integral to the Fund’s culture, strategic planning, decision making and resource allocation” [30]. The report highlighted the organization’s “three lines of defence,” operations, oversight, and independent auditors—including the OIG [31]. Notably, risk oversight has become a standing topic for the Board and its committees [31].

Overall, the Global Fund’s risk management framework has produced results. In 2017, risks in the categories of “Grant-Related Fraud & Fiduciary,” “Procurement,” and “Quality of Health Products,” decreased from high classification to moderate, resulting in “a much lower pace of OIG and non-OIG identified recoverables, and fewer issues related to procurement” [34].

### Internal and external institutional oversight

#### Ethics and integrity framework at the Global Fund

The Global Fund developed their Ethics and Integrity Framework in 2014, which was followed by the establishment of an Ethics Office in 2016 to provide advice, manage conflicts of interest, and promote ethics and integrity through trainings, presentations and workshops [27, 35]. According to Transparency International, ethics is defined as “*a set of standards for conduct in government, companies and society that guides decisions, choices and actions*,” and having a strong ethics and integrity framework allows an organization to be more accountable and transparent [36]. Since its creation, the Ethics Office has become increasingly engaged. This is evidenced by the increase in 2018 to 245 cases processed related to ethics, misconduct, and conflict of interest, compared to the 183 cases in the previous year [35]. There was also an increased number of preventative actions taken, which the Ethics Officer interpreted as stakeholders progressively “considering ethics and integrity and seeking to take trusted decisions in the interest of our mission” [35].

Conflict of interest is defined by the Global Fund as, “*a situation in which a[n] official has a competing professional or personal financial interest that could have real or perceived effects on the official’s ability to fulfill his/her responsibilities with the best interests of the Global Fund in mind*” [37]. Conflicts of interest are also considered a serious gateway to corruption in the health system. They can be financial or non-financial and can pertain to institutions or individuals—with the latter having the potential to lead to institutional corruption [38]. Good practices to manage conflicts of interest in international organizations include attention to ACTA mechanisms, conflict of interest disclosures and reporting, independent monitoring, and sanctions when conflicts are present, such as abstention or withdrawal from decision-making processes [39]. While many institutions have policies to disclose conflicts of interest, without sanctions in place, these policies do not always decrease corruption and increase transparency.

Conflict of interest is a focus of the Ethics Office, and in 2018, 177 conflict of interest cases were identified, of which 82 cases were cleared; 73 cases had “mitigating measures” put in place; and 17 cases remain uncleared [35]. Notably, for the individuals or institutions whose conflict of interest was not cleared, they were either unable to take the position or assignment or had to step down from their position [35]. Within the Global Fund, there has also been significant improvement in reporting conflicts of interest. For example, only 76% of Board members completed “Declarations of Interest” in 2014, compared to the 100% completion rate in 2019 [27]. In 2018, the Ethics Office processed declarations of interest from 836 people [31]. To streamline declarations, the Ethics Office has planned to implement an automated, centralized system to declare conflicts of interest from June 2019 [27].

#### The Office of the Inspector General

While the aforementioned ACTA mechanisms were implemented from 2011 onwards, the OIG was established in 2005 and is the main unit that conducts audits of the Global Fund’s activities and investigates alleged prohibited practices according to its “Policy to Combat Fraud and Corruption” [20, 27, 40]. Its purpose is “to expose the abuse of grant funds” and implement the organization’s whistleblowing policy and annual self-assessments. The OIG is also subject to external assessments every three years to maintain accountability and transparency [27, 41–43]. While the OIG is considered “the only risk-mitigation strategy within the Global Fund that has worked as designed” [5], it is included in the scope of this paper as constant improvements and new initiatives have been made since 2011, such as the I Speak Out Now! campaign.

The OIG’s external assessments have been largely favourable and its latest 2017 external assessment noted that “there has been no degradation from the 2014 scorings, with progress being made” [41]. In addition, the report noted that the “OIG investigation function has clearly demonstrated its keen appetite for constant improvement” from its twice-monthly “Innovation and Ideas” meetings [41]. Over the years, the OIG has become more proficient and has significantly improved its average time to close investigations from 7.6 years in 2005 to 2.5 years in 2010, and then to 7.9 months in 2015 [44].

#### OIG audit and investigation reports

In 2008, the OIG began publishing comprehensive audit and investigation reports on its website to increase transparency, and as of April 7, 2020, there were a total of 145 internal and country audit reports and 58 investigational reports posted. Each report makes specific recommendations on how to mitigate fraud and corruption [40, 45]. The OIG audit and investigation reports have been an efficient means to increase transparency and accountability within the Global Fund, as demonstrated by the OIG country audit reports that led to the HLP recommendations and subsequent implementations of ACTA throughout the organization.

Agreed Management Actions (AMAs) are published in the audit and investigation reports and are defined as “*an agreed course of action, decided jointly between the Secretariat and the Office of the Inspector General, to remedy an identified root cause, targeting specific portfolios where progress is needed*” [46]. The Global Fund tracks AMA progress in its “Joint AMAs Progress Reports” to ensure there is follow-up [46]. In November 2018, the Global Fund demonstrated “significant progress” through its all-time low numbers of 68 open AMAs and 22 overdue AMAs [46]. The 2019 Joint AMAs Progress Report listed 78 open AMAs and 13 overdue AMAs as of August 2019 [47].

### Novel initiatives

#### I speak out now! Campaign

Whistleblowing is identified as an important pathway to report and expose corruption within an organization [48]. It has been defined as “*an open disclosure about significant wrongdoing made by a concerned citizen totally or predominantly motivated by notions of public interest*,” and can lead to increased perceived transparency [49]. When institutions fail to prevent disasters, whistleblowers ring the alarm, often prioritizing the public good over their own personal safety [50]. People who blow the whistle on malpractice or corruption in their workplace often do so in line with organizational policies, yet they commonly experience adverse consequences and can be treated as dissenters by colleagues and higher management [50]. Whistleblowing is an effective anti-corruption tool within an international organization, therefore encouraging whistleblowers to speak out against malpractice, and protecting them from reprisals taken against them, can increase transparency and accountability.

Accordingly, the Global Fund has developed a grassroots project to further strengthen its ACTA mechanisms, and on International Anti-Corruption Day, December 9, 2015, the OIG launched the *I Speak Out Now!* campaign to encourage Global Fund staff and grant implementors to report fraud, corruption, and human rights violations [51, 52]. The OIG rolled out the e-learning platform to help educate staff and grant implementors on the early signs of corruption and fraud, and to provide a channel for whistleblowing [53]. In addition, the OIG developed an “Anti-Corruption and Anti-Fraud Tool Kit” which it distributes to its Principal Recipients (PRs) and CCMs [54]. The tool kit is available in multiple languages and contains an anti-fraud and corruption self-assessment; the Global Fund’s whistleblowing policy; a corruption reporting template; and an incident management spreadsheet amongst other tools [54].

In addition, the OIG collaborated with Malawi, Côte d’Ivoire, and Ukraine to pilot anti-fraud and anti-corruption materials to address drug theft, diversion, and bribery through the *I Speak Out Now!* campaign [51, 53]. For example, in Malawi, anti-fraud and anti-corruption flyers were distributed to suppliers, announcements were made on the radio, a local hotline was set up, and an anti-malarial drug theft task force was established [53]. Over 100 whistleblower reports were received at the hotline within a few months, resulting in several arrests, fines, and prosecutions [53]. The OIG also noted that more people were reporting allegations, with 18% more reports from January to May 2018 than for the same period in 2017 [55]. The OIG also has created formal classroom training for country implementors and the Secretariat to raise awareness and to educate on evidence of fraud and effective strategies to mitigate risks [55]. The *I Speak Out Now!* campaign has so far been effective, with the OIG’s external assessment report calling it “impressive” and “well executed” [41].

#### Reducing procurement fraud through pooled procurement

Procurement is known to be one of the most vulnerable areas within the health system to corruption due to the substantial financial rewards offered by large procurement contracts as well as its technical complexity [11]. When present in procurement processes, corruption can result in drug shortages, inflated prices of medicines, and an influx of substandard and falsified medicines—all undercutting public health goals. Corruption commonly emerges in pharmaceutical procurement systemically or as isolated incidents and manifests throughout procurement tendering [11]. During the pre-bidding, bidding, and post-bidding phases of tendering, risks of corruption include single individuals posing as multiple bidders, falsified documents, and bribery [11]. ACTA mechanisms are essential in procurement processes, as a lack of controls is shown to contribute to procurement corruption [11]. Reversely, when corruption is controlled, good pharmaceutical procurement can increase access to medicines and support sustainable development goals [11].

As the Global Fund’s spending on procurement makes up 10% of the global market for public health products [56], procurement fraud has traditionally been a significant challenge for the organization [57]. At the time of the HLP report, the Global Fund had a Voluntary Pooled Procurement (VPP) program in place. The HLP recommended that pooled procurement be required, and that drug storage and delivery be outsourced if the Global Fund did not find local structures to be adequate [5]. These two recommendations were heavily criticized by Aidspan, an NGO and watchdog organization that actively highlights, analyzes and influences the Global Fund’s transparency and effectiveness [58]. Aidspan disagreed with the HLP’s opinion that the VPP was effective because there were reports that it was becoming slower and “smaller countries with small orders did not get good service” [59]. Aidspan and members of the Global Fund also rejected the Panel’s recommendation to outsource storage and delivery because it would undermine country ownership and national capacity building [59, 60]. The HLP later retracted this recommendation, but the Global Fund continued to expand the VPP, recognizing the importance of procurement [59].

The Global Fund’s Pooled Procurement Mechanism (PPM) evolved from the VPP and was designed to improve procurement quality, decrease prices, and reduce procurement fraud [35, 57, 61, 62]. The Global Fund combines procurement orders from implementing countries to increase negotiating power, which helps to reduce prices and to ensure quality [63]. In 2016, the Global Fund also launched a new online procurement platform called wambo.org, which was designed to increase transparency, improve product availability, reduce costs, and promote local capacity building. It also creates sustainable supplies by allowing country implementors to compare prices, lead time, and the quality of products [63, 64]. The Global Fund now states that it may require implementing countries to use the PPM if that country’s procurement and supply management capacity are inadequate [62].

In 2017, the OIG published an audit report of wambo.org and stated that it had “brought increased transparency to the ordering process” and that clients were very satisfied with the platform [63]. The vision of wambo.org was to be a “self-sustaining global public good allowing countries to place orders using domestic funding and offering global and transparent pricing for all stakeholders.” In May 2017, the Board accepted a pilot program for wambo.org to be used by countries with domestic funds [63]. Subsequently, in 2019, the Board expanded wambo.org to “be made available for non-Global Fund-financed orders by governments and non-government development organizations in Global Fund-eligible and transitioned countries, for all products, services and functionalities as they become available on wambo.org” [65]. This marks a significant change in the evolution of wambo.org because it is now available to a broader market.

As a result of the PPM, data shows trends of fraud and corruption within the Global Fund have shifted away from largely procurement fraud in 2014–2015 to a diversity of fraud and corruption [43, 57, 61]. This can be seen in the OIG’s 2018 Annual Board Report, which demonstrates a reduction from 80% of investigations relating to procurement fraud between 2014 and 2015, to 20% of investigations in 2018 [43]. Three areas of particular concern to the OIG were the increasing number of drug thefts from storage facilities; the emergence of data fraud, where data for Global Fund resource-funded surveys were falsified; and “salary kickback schemes,” where a fund recipient’s senior management collects a percentage of an employee’s salary as their own [43].

## Discussion

The Office of the United Nations High Commissioner for Human Rights emphasizes that corruption is a “major obstacle to the effective protection of human rights,” and has stated that transparency, accountability, and meaningful participation are effective means to fight corruption [66]. Global institutions such as the UNDP, the World Bank and the WHO are focusing on building an evidence base on the tools to best control corruption and thus contribute to the SDGs, specifically SDG 3 “Good Health and Well-Being” and SDG 16 “Peace, Justice and Strong Institutions,” which includes the sub-target 16.5 “(to) substantially reduce corruption and bribery in all their forms” [32]. There is a veritable and urgent need for novel academic research on which international institutions and instruments have worked best to control corruption and why. Despite the increasing efforts of international institutions to address the issue of corruption, an important question remains that is relevant to policy and operations which global development agencies do: how do the distinctive hard and soft law features of leading global governance institutions affect their understanding of and approach to anti-corruption instruments, and the effective implementation and outcomes of those instruments?

There are considerable challenges to quantifying the costs of corruption in global health as it is often hidden and can go unreported, however, estimates put it in the billions of dollars [67]. More narrowly, in terms of human development, corruption has a significant, negative effect on health indicators such as infant and child mortality, even after adjusting for income, female education, health spending, and level of urbanization [68, 69]. Corruption hurts economic and human development and has the harshest impact on poor and marginalized populations. Thus, global institutions have begun to discuss corruption, and in the past decade have launched programmes to control it in innovative ways, such as through designing anti-corruption tools that also contribute to good governance.

The effect of ACTA mechanisms on local governance is an area where further research is needed. In 2011, the HLP identified that when implementing countries truly took hold of the Global Fund’s idea of “country ownership,” good governance also improved. According to Kavanagh et al. (2019), the Global Fund improved the control of corruption, regulatory quality, voice and accountability, rule of law, human development, and total adult mortality when greater financing was provided to fight diseases through the unique mechanisms of the Global Fund [70]. This could suggest that the benefits of ACTA mechanisms in health systems have positive effects on governance within a country however, further research is required.

The Global Fund has worked to advance its “zero tolerance to corruption” policy and adopted measures to enact ACTA within the organization, particularly from 2011 onwards with the exception of the OIG which has since implemented significant improvements. As an international organization, the Global Fund is required from a fiduciary and a public relations standpoint, to disclose how they implement accountability and transparency within the institution. As we have demonstrated in this paper, the Global Fund has a wide range of mechanisms designed to promote accountability and transparency. Less apparent*,* however, is how effective these various mechanisms are towards achieving institutional goals.

In addition, there has been increasing public scrutiny on international organizations during the past two decades, which the HLP identified as three global trends of “increasing financial austerity in global health,” “rising standards for accountable use of development resources,” and “increased demand for precision in the measurement and reporting of results” [5]. The ACTA mechanisms implemented within the Global Fund were accelerated by public attention resulting from the AP article in 2011, and the numerous prior warnings from scholars and academics. For this reason, a robust evaluation of the impact of accountability and transparency mechanisms within international organizations is warranted; however, these are not easy to evaluate given that there is no common standard of transparency or accountability against which international organizations can be evaluated [71]. What is more, evaluation processes in international organizations lack necessary techniques and methodologies and are “fragmented, non-comprehensive and non-integrated” [72, 73]. Of equal importance, international evaluations are often internal and devoid of critical independent reviews [74–76]. As the Global Fund leads global partnerships against corruption with the WHO and the UNDP, it will become increasingly necessary to develop tools to assess ACTA mechanisms within international organizations and determine their impact.

Understanding the outcomes of ACTA mechanisms within international organizations is important, as potential perverse impacts can accompany corruption controls. For example, one downside of ACTA mechanisms is the increased requirements and burden on local health systems, as experienced by local Global Fund staff. In the District Level Network (DLN) offices set up locally as part of the Global Fund’s grants, the “excessive burden” of paperwork was a common complaint resulting from enhanced reporting mechanisms on top of the already heavy workload [1]. DLN staff are responsible for maintaining reports of all interactions and activities, along with a “record of every individual counselled, all support meetings organised, peer educator trainings held, each person followed up on treatment, peer educator’s working registers and other similar records. This information is then entered into cumbersome computer databases accessible to the state and national network office” [1]. Additionally, participation in the organization’s programmatic reorientation programs has resulted in multiple majority staff absences, leaving few DLN staff to manage records and reporting, often unable to complete their own work tasks [1]. Further indication on the effectiveness of such programs is needed to understand any possible perverse impacts of ACTA controls, specifically regarding the impact of increased burden on local staff and health systems.

## Conclusions

ACTA mechanisms are necessary to enhance efficiency in health systems and ultimately ensure that health financing and resources fulfill their intended purposes. To date, ACTA efforts in health have been largely disconnected, with major target reductions in global health sector corruption unachieved. Up to this point, ACTA initiatives in the health sector have been plagued by fragmentation and unable to deliver a coordinated response to corruption.

To address these issues, the Global Fund has joined other international organizations, such as the WHO, the UNDP, and the World Bank to set up the Coalition for Accountability, Transparency, and Anti-Corruption in Health (CATCH). CATCH is a multi-stakeholder and multi-sectoral initiative aimed at strengthening global health outcomes through the prevention of health-sector corruption. While CATCH is still in its infancy, progress on the alliance’s initiatives is unreported, however, it marks a step in the right direction as a much-needed global health initiative that applies a sectoral approach to addressing corruption.

From 2011 onwards, the Global Fund has made efforts to implement ACTA in the organization and to mitigate fraud and corruption in its operations. These internal mechanisms have particularly reduced grant-related risks and procurement fraud as demonstrated by the decreased classification from high to moderate in 2017, and the reduction of investigations in 2018 respectively. Additionally, through the employment of mechanisms such as the *I Speak Now!* campaign, the Pooled Procurement Initiative, and the AMAs, the Global Fund has displayed a commitment to addressing corruption risks within their operations and at the country level. However, as is demonstrated in this paper, there is a lack of significant evidence on the effectiveness of the implemented ACTA mechanisms and institutions. There is a potentially urgent need to evaluate both internal and external ATCA mechanisms and institutions in place to ensure that the intended impacts are being achieved. To do this, it is first necessary for the Global Fund and other international organizations to define and clarify ACTA concepts and their impacts. While much progress in this area has been made, it remains critical that moving forward the Global Fund continues to monitor their ACTA mechanisms and institutions and take further steps to ensure their efficacy.

### Limitations

This study was limited to a document analysis in the English language only. Results of the document analysis were not validated by key informants within the Global Fund. Further research can be done by conducting key informant interviews to validate the results of this study and to better understand the impact of the Global Fund’s ACTA mechanisms. Additionally, this study heavily relied on Global Fund published documents and peer-reviewed articles and did not include local perspectives or media reporting beyond the Associated Press article.

## Data Availability

Not applicable.

## Abbreviations

ACTA: Anti-corruption, transparency, and accountability
AMA: Agreed management action
AP: Associated press
CATCH: Coalition for accountability, transparency, and anti-corruption in health
CCM: Country coordinated mechanism
CRO: Chief risk officer
DLN: District level network
HLP: High-level independent review panel
OIG: Office of the inspector general
PPM: Pooled procurement mechanism
PR: Principal recipient
SDG: Sustainable development goal
UNDP: United Nations development programme
UNODC: United Nations office on drugs and crime
VPP: Voluntary pooled procurement
WHO: World health organization
